# 

**Published:** 1856-06

**Authors:** 


### PROSPECTUS.

### THE DENTAL REGISTER OF THE WEST,

During the past nine years of its existence, has made its acquaintance with the profession generally. Of its past career we need say but little, for it has spoken for itself. Of its future, we will be allowed to express our hopes and intentions. It began its existence with forty-eight pages, and in obedience to the calls of professional progress, has grown to its present size. If our professional brethren are willing, we intend that its future shall correspond with its past growth. That willingness can be best manifested by aiding us in filling its pages with useful matter and in extending its circulation so that we can afford to enlarge it at the present price.

The *Register* will be issued, promptly, on the first of October, January, April and July; and, as formerly, will be devoted solely to the cause of Dental Science.

The contents of the *Register* will be arranged under the following divisions:

1. Original Lectures, Essays, &c.
2. Selected Articles.
3. Correspondence and Proceedings of Societies.
4. Reviews and Notices.
5. Miscellaneous Items.
6. Editorial Department.

In entering our new field of labor we feel determined that no effort shall be spared to keep the readers of the *Register* informed of professional improvements and interesting cases, and we trust that our want of experience will be counterbalanced by the time and attention our *united* effort will enable us to give it. Having, also, the promise of the advice, assistance and co-operation, of the retiring editor, from whose pen we expect frequent communications, we hope that neither the profession nor the *Register* will suffer by the change.

Contributions to the pages of the *Register* are respectfully solicited. We wish to give the original department greatly the predominance.

Terms:—Single copy $3 per annum, in advance, two copies $5.

All communications should be addressed, "Dental Register," or Taft & Watt, Cincinnati, O.

### A. J. WATTS' IMPROVED PREPARED GOLD.

By a course of continual experiments, and conference with the Profession, as to its needs, this article has recently been brought to a degree of perfection which renders it indispensable to every operator who becomes in the least experienced in its use. Its adhesive and plastic quality, and perfect freedom from crumbling, now leave nothing to be desired in this respect.

Since its first introduction to the Profession, it has been very generally, and, in many instances, exclusively adopted by the first operators, whose continued experience demonstrates,

1st. That it requires for its successful use peculiar tools, combined with a peculiar system of manipulation.

2d. That it requires skill to use it, repaying even the highest degree, by producing results heretofore unparalleled in beauty and permanency.

3d. That after proper experience on the part of the Operator, it may be used in *all* cases where foil could be used, and in many cases where foil could not be used at all; and in every instance with the most complete success.

4th. That after all the changing influences of time, the action of the fluids, or secretions of the mouth, the effects of heat and cold, or the continual friction of mastication, stoppings properly made of the Watts' Gold remain in all respects *permanent in their character.* They do not absorb the fluids, nor break up, disintegrate, soften, dissolve, become porous, or in any respect change their nature or condition.

5th. That by it a large and important class of frail teeth, hitherto deemed beyond the reach of art to save, are restored to health and permanent usefulness.

6th. It has been demonstrated by absolute experiment that the Watts' Gold, which is necessarily *pure*, may be so consolidated in the process of filling as to be one-fourth harder than melted gold, or the most skillfully packed Gold Foil, and even harder than twenty carat condensed gold coinage.

7th. It clearly demonstrates that its use will open a new and inviting field for skillful enterprise; and while it calls out and cultivates a higher and continually augmenting skill, it abundantly repays with an ever-increasing ratio of success, producing results hitherto unattained, or even anticipated, by the Profession.

It is put up in one-eighth ounce boxes, containing directions for its use, and can be mailed to any part of the world.

PRICE, RETAIL, $32. ONE OUNCE OR MORE, AT $30 PER OUNCE. Address, A. J. WATTS & CO.,

*July*, 1855. UTICA, NEW YORK

